# Identification and gene expression analysis of cytosine-5 DNA methyltransferase and demethylase genes in *Amaranthus cruentus* L. under heavy metal stress

**DOI:** 10.3389/fpls.2022.1092067

**Published:** 2023-01-04

**Authors:** Veronika Lancíková, Jana Kačírová, Andrea Hricová

**Affiliations:** Institute of Plant Genetics and Biotechnology, Plant Science and Biodiversity Centre, Slovak Academy of Sciences, Nitra, Slovakia

**Keywords:** *Amaranthus cruentus*, methyltransferase, demethylase, heavy metals, gene expression

## Abstract

Amaranth has become increasingly popular due to its highly nutritious grains and ability to tolerate environmental stress. The mechanism underlying defense and adaptation to environmental stress is a complicated process involving DNA methylation and demethylation. These epigenetic features have been well documented to play an important role in plant stress response, including heavy metal-induced stress. This study was aimed at the identification and analysis of cytosine-5 DNA methyltransferase (*C5-MTase*) and demethylase (*DMTase*) genes in *Amaranthus cruentus*. Eight *C5-MTase* and two *DMTase* genes were identified and described in response to individual heavy metals (Cd, Pb, Zn, Mn) and their combination (Cd/Pb, Cd/Zn, Pb/Zn) in root and leaf tissues. Studied heavy metals, individually and in combinations, differentially regulated *C5-MTase* and *DMTase* gene expression. Interestingly, most of the genes were transcriptionally altered under Zn exposure. Our results suggest that identified amaranth *MTase* and *DMTase* genes are involved in heavy metal stress responses through regulating DNA methylation and demethylation level in amaranth plants.

## 1 Introduction

DNA methylation is defined as a conserved and heritable epigenetic modification that plays an essential function in the regulation of plant development and responses to stress conditions (Zhang and Lang, 2018; Gallego-Bartolomé, 2020). The most common and the best characterized epigenetic modification is the 5-methylcytosine (5-mC) which possess a methyl group at the 5-carbon position of cytosine (Pavlopoulou and Kossida, 2007; Harrison and Parle-McDermott, 2011; Zhang and Lang, 2018). DNA methylation in plants is found in both symmetric (CG and CHG) and asymmetric (CHH) contexts, where H = A, T, or C (Chen and Li, 2004; Bartels et al., 2018). The level of cytosine methylation in plant genome is different and not stable. In *Arabidopsis*, genome-wide methylation mapping has shown that 20–33% of genes are methylated (Penterman et al., 2007). In rice genome, 13–15% of the total cytosines are methylated, and their number increases under stress (Kumar et al., 2022). In higher plants, the level of DNA methylation is controlled by three mechanisms: initiation of DNA methylation *de novo*, maintenance of pre-existing DNA methylation and DNA demethylation (Wang et al., 2016; Bartels et al., 2018; Yu et al., 2021). Based on the data obtained in *Arabidopsis* and other plant species, DOMAINS REARRANGED METHYLTRANSFERASE 1 and 2 (DRM1 and DRM2) initiate *de novo* DNA methylation (Cao and Jacobsen, 2002; Zhang, 2006; Bartels et al., 2018), DNA METHYLTRANSFERASE 1 (MET1) maintains CG methylation (Finnegan and Dennis, 1993; Kankel et al., 2003) and CHROMOMETHYLASE 2 and 3 (CMT2 and CMT3) primarily maintain CHG and CHH DNA methylation (Bartee et al., 2001; Lindroth et al., 2001; Du et al., 2012; Wang et al., 2016). DNA methylation is a reversible modification, thus DNA demethylases DEMETER (DME), DEMETER-like 2 (DML2), DEMETER-like 3 (DML3) and REPRESSOR OF SILENCING 1 (ROS1) actively remove 5-mC through the base excision repair pathway (Zhu, 2009; Bartels et al., 2018; Liu and Lang, 2020).

To a large extent, DNA methylation regulates plant growth and development through a cascade of gene repression and activation (Zhang and Lang, 2018; Kumar and Mohapatra, 2021). Abiotic and biotic stresses can trigger changes in DNA methylation at the level of individual loci or whole-genome (Liu and He, 2020). Epigenetic stress memory might be a crucial part of plant defense strategy to cope with extreme and inevitable environmental changes (Kinoshita and Seki, 2014; Kumar et al, 2016; Kumar, 2018; Ashapkin et al., 2020). Land pollution from rapidly expanding industrialization negatively influences the crop growth and production worldwide. Thus, knowledge of processes associated with abiotic stress and identification of the factors responsible for stress memory can provide an opportunity to improve plant tolerance.

Grain amaranths, namely *Amaranthus cruentus*, A. *hypochondriacus*, and *A. caudatus* are highly nutritional pseudocereals characterized by the ability to tolerate environmental stress (Coelho et al., 2018; Lancíková et al., 2020; Pulvento et al., 2022). There is still a little known about the molecular mechanisms responsible for such a strong stress tolerance, and even less is known about epigenetic regulation in *Amaranthus*. Herein, we aimed to identify cytosine-5 DNA methyltransferase (*C5-MTase*) and demethylase (*DMTase*) genes in amaranth. Specific genes encoding amaranth DNA MTases and DMTases have not been previously identified and/or analyzed. Furthermore, the transcript abundance was analyzed in root and leaf tissues under the normal growing conditions and also in response to heavy metal (HM) stress. Specifically, the effect of cadmium (Cd), lead (Pb), zinc (Zn) and manganese (Mn) on the activity of *C5-MTase* and *DMTase* genes was tested. The effect of HMs was tested individually and also in the following combinations Cd/Pb, Cd/Zn and Pb/Zn. Understanding of epigenetic regulation in amaranth plants might provide an essential information how crops are dealing with HM pollution.

## 2 Materials and methods

### 2.1 Plant material cultivation and heavy metal treatments

Amaranth (*Amaranthus cruentus* L.) variety “Pribina”, previously bred and registered in Slovakia, was used for the analysis. Amaranth plants were cultivated as previously described by Lancíková et al. (2020). Briefly, the hydroponic experiments were performed in the growth chamber at 23°C, 16/8 light/dark cycle and 50% humidity (KK 1450 TOP+FIT model, POL-EKO Aparatura, Poland). Amaranth was germinated and cultivated in soil until the stage of 4-5 true leaves, then transferred into hydroponic solution (Hoagland and Arnon, 1950). To study the effect of HMs, amaranth was cultivated in clean Hoagland solution for one week, consequently HMs were added into solution. Plants were cultivated with HMs for two weeks, control plants were cultivated alongside. Then, root and leaf tissues were collected for analysis. HMs were added into hydroponic solution either individually, thus Cd (CdCl_2_; 15 mg.L^-1^), Pb (PbNO_3_; 200 mg.L^-1^), Zn (ZnCl_2_; 150 mg.L^-1^), Mn (MnCl_2_; 300 mg.L^-1^) or in combinations [Cd/Pb (15 + 200 mg.L^-1^), Cd/Zn (15 + 150 mg.L^-1^) and Pb/Zn (200 + 150 mg.L^-1^)].

### 2.2 Identification and characterization of *A. cruentus* DNA *MTase* and *DMTase* genes

For *in silico* identification of MTase and DMTase, the genome of *Amaranthus hypochondriacus* was retrieved from the online Phytozome database (https://phytozome-next.jgi.doe.gov/info/Ahypochondriacus_v2_1). To search C5-MTase and DMTase protein sequences, the conserved key domains were employed as queries. The Hidden Markov Model (HMM) was obtained from PFAM database (http://pfam.xfam.org/). The HMM ID of C5-MTase conserved key domain is PF00145 and the HMM IDs PF00730 and PF15628 are for DNA DMTase conserved key domains. The candidate proteins were confirmed and classified with the Simple Modular Architecture Research Tool (SMART, http://smart.embl-heidelberg.de/) (Schultz et al., 1998). Incomplete and redundant protein sequences were removed. The ExPASy tool (https://web.expasy.org/protparam/) was used for calculating the grand average of hydrophobicity (GRAVY), molecular weight (MW) and isoelectric point (pI). The subcellular localization was predicted with Plant-mPloc (http://www.csbio.sjtu.edu.cn/bioinf/plant-multi/) (Chou and Shen, 2010). Prediction of nuclear localization signals for identified C5-MTase and DMTase proteins was performed using cNLS Mapper (https://nls-mapper.iab.keio.ac.jp/cgi-bin/NLS_Mapper_form.cgi) (Kosugi et al., 2009).

### 2.3 Conserved motifs, gene-structure, protein-protein interaction analysis

Full length amino acid sequences of amaranth C5-MTases and DMTases were used for conserved motif analysis using Multiple Expectation Maximization for Motif Elicitation (https://meme-suite.org/meme/) (Bailey et al., 2015) software version 5.4.1. Genomic and coding sequences of C5-MTases and DMTases in amaranth were analyzed using Gene Structure Display Server (http://gsds.gao-lab.org/) (Hu et al., 2015) version 2.0, and schematic diagrams of individual genes were displayed. The protein-protein interaction network was constructed using the STRING (https://string-db.org/) (Snel et al., 2000) software version 11.5.

### 2.4 Phylogenetic analysis

Amaranth C5-MTase and DMTase protein sequences were aligned by Molecular Evolutionary Genetics Analysis (MEGA X) (Kumar et al., 2018) software version X. Subsequently, an evolutionary analysis based on the Neighbor-Joining method with 1000 bootstrap replicates was conducted using MEGA X.

### 2.5 RNA isolation and gene expression analysis

RNA isolation, reverse transcription and quantitative PCR were performed as previously described (Lancíková et al., 2020). In summary, total RNA was extracted according to the protocol based on TriZOL reagent (Chomczynski and Mackey, 1995). Approximately 50 mg of plant tissues were incubated with TriZOL, then chloroform extraction was performed and supernatant was precipitated using isopropanol as previously described (Lancíková et al., 2020). The cDNA was synthetized from 1 µg of RNA using Maxima First Strand cDNA Synthesis Kit for RT-qPCR (Thermo Fisher Scientific, Waltham, USA) according to the manufacturer’s instructions.

Transcript abundance of identified *C5-MTase* and *DMTase* genes was evaluated using qPCR according to the previously published protocol (Lancíková et al., 2020). Briefly, standard curves were generated for qPCR optimization using the series of five-fold cDNA dilutions (1:1, 1:5, 1:25, 1:125, 1:625, and 1:3125). For each gene, PCR efficiency (E), and correlation coefficient (*R* ^2^) were determined using the linear regression. PCR efficiency of 90-110% and *R* ^2^>0.99 were accepted. The quantitative PCR (qPCR) was performed in the LightCycler^®^ Nano (Roche, Basel, Switzerland). The reaction mixtures consisted of 2x SsoAdvanced Universal SYBR^®^ Green supermix (Bio-Rad, Hercules, USA), 400 nM of each forward and reverse primer, 50 ng of cDNA, and nuclease-free water added up to the total reaction volume of 10 µl. Two-step amplification protocol was applied, initial denaturation at 95°C for 30 sec; 45 cycles of denaturation at 95°C for 15 sec and annealing/polymerization at 60°C for 60 sec; then melting analysis from 60°C to 97°C at 0.1°C/s was performed to verify the specificity of the desired amplicon. Expression of all analyzed genes was determined in each reaction using the threshold cycle (Ct value). The Ct value was set automatically by LightCycler Nano software. Calculation of relative gene expression was performed according to Pfaffl (2001) using the PCR efficiencies and Ct values of control and unknown samples. The *Amaranthus hypochondriacus Tubulin* (*AhTUB*) gene was used as an internal standard in all experiments. The primer sequences of the MTases, DMTases and reference gene are shown in the **Table 1**.

**Table 1:** Primer sequences for analyzed *MTase*, *DMTase* and reference genes in *A. cruentus*.

GENE	FORWARD 5’-3’	REVERSE 5’ 3’
*AcMET1a*	GTGGTTTTGGAGAACTTGGGG	GCATGCTTACGTGACTGGGA
*AcMET1b*	GGCCAATGGGGAAAATGCTT	CGATCGCAGCTATCTCGCTT
*AcCMT1*	CAAAGCAAACCAAGTCGGGG	CTCCAGCTAAGGTTGCACCA
*AcCMT2a*	CTTCCTCCGGTGACAAACGA	AACATCCGGAGAACCCAACA
*AcCMT2b*	TGGGGGAAAATGCGTCGTTA	TATCGCCAAAGACGAAGGCA
*AcCMT3*	CAAACTGTGGGGTCGGAGTT	TTGTTGTCAGCACGAACACG
*AcDRM2a*	CCTGGTCCGGTATCAGAGGA	GACGCCCTTGCCATTTGTG
*AcDRM2b*	CAGAGGCTTCAATCTGGCGA	GCCACCACCTCTTGTGTGAT
*AcDML2a*	GTCCATTCACAGCCTGACCA	CTCTGCTGTTGCTCACTGGA
*AcDML2b*	TGACCATCCACTGCTCAAGG	TCGCGGTTCGAACAGGTATC
*AhTUB*	TCTCAGCAGTATGTCTCCCTC	TCTACTTCTTTGGTGCTCATC

### 2.6 Statistical analysis

Statistical analysis of the obtained data was performed using the GraphPad Prism version 9.4.0. (GraphPad Software, Inc., San Diego, USA). All analysis were performed in three biological replicates. Statistical significance was analyzed using the one-way ANOVA with *post-hoc* Tukey’s multiple comparisons test.

## 3 Results

### 3.1 Identification, structural analysis, nuclear localization of *Amaranthus cruentus* MTase and DMTase

Taken together, eight MTases and two DMTases were described in *Amaranthus cruentus*. Based on the identified conserved domains, these DNA MTases were divided into three groups – MET1, CMT and DRM2. MTases and DMTases were named according to the highest identity score with their closest homolog as follows: two members of MET1 group, AcMET1a and AcMET1b, four members of CMT group, AcCMT1, AcCMT2a, AcCMT2b and AcCMT3, two members of DRM2 group, AcDRM2a and AcDRM2b, and two DML2 DMTases, AcDML2a and AcDML2b.

Key DNA methylase conserved domains were identified, namely bromo adjacent homology (BAH) domain and replication foci domain (RFD) in MET1 group and BAH and chromo (Chr) domains in CMT group. MET1s are homologs of mammalian DNMT1, while CMTs are plant-specific DNMTs. Two identified DMTases belong to the DML2 group, and harbored RNA recognition motif-DME (RRM-DME) domain, permuted single zf-CXXC (Perm-CXXC) domain and endonuclease III (ENDO3c) domain. Structural analysis showed that AcCMT1, AcCMT2a and AcCMT2b harbor one BAH domain and one Chr domain, while AcCMT3 harbors one BAH domain and two Chr domains. MET1 was characterized by presence of two RFD and two BAH domains.

The length of amino acid (AA) sequences varied from 256 (AcMET1a) to 1492 (AcMET1b) in C5-AcMTase proteins. The AA sequences length of DMTase was 1929 and 286 for AcDML2a and AcDML2b, respectively. The molecular weight ranged from 28.43 to 215.08 kDa with a pI 4.76-7.79, mostly indicating the neutral and basic nature of the identified proteins. The GRAVY index of the identified proteins varies from the lowest -0.576 (AcDRM2b) to the highest -0.154 (AcMET1a), thus *A. cruentus* C5-MTases and DMTases are hydrophilic. The predicted subcellular localization for the most identified proteins was in the nucleus, except the proteins AcMET1a and AcDRM2b localized in chloroplast (**Tables 2**, **3**).

**Table 2:** Characterization of *A. cruentus C5-MTase* and *DMTase* genes; ^1^Gene ID from *A.hypochondriacus* genome in Phytozome database; ^2^Chromosome location in the genome of *A.hypochondriacus*; ^3^AA – number of amino acids; ^4^MW – protein molecular weight; ^5^GRAVY – grand average of hydrophobicity; ^6^pI – isoelectric point; ^7^Intron number; ^8^Predicted subcellular localization by Plant-mPloc software.

Gene name	Gene ID^1^	Chromosome localization^2^location	AA (aa)^3^	MW (kDa)^4^	GRAVY^5^	pI^6^	Intron^7^	Predicted subcellular localization^8^localization
*AcMET1a*	AH001971-RA	Scaffold_1:32547615.32563037	256	28.43	-0.154	7.79	3	Chloroplast
*AcMET1b*	AH006822-RA	Scaffold_4:3862657.3871287	1492	167.52	-0.488	5.45	11	Nucleus
*AcCMT1*	AH003426-RA	Scaffold_2:16586229.16601252	803	91.11	-0.53	5.39	20	Nucleus
*AcCMT2a*	AH007324-RA	Scaffold_4:13151061.13170913	1339	149.58	-0.457	4.98	19	Nucleus
*AcCMT2b*	AH018462-RA	Scaffold_12:5201808.5215197	988	111.44	-0.492	5.65	20	Nucleus
*AcCMT3*	AH010889-RA	Scaffold_6:20787067.20799928	1325	148.75	-0.561	4.82	29	Nucleus
*AcDRM2a*	AH015836-RA	Scaffold_10:13471627.13480075 reverse	527	58.38	-0.445	4.76	9	Chloroplast/Nucleus
*AcDRM2b*	AH004791-RA	Scaffold_3:1047582.1051004 forward	472	53.26	-0.576	6.27	6	Chloroplast
*AcDML2a*	AH012665-RA	Scaffold_8:2344820.2356763 reverse	1929	215.08	-0.721	6.38	18	Nucleus
*AcDML2b*	AH023483-RA	Scaffold_16:11334720.11349586	286	32.51	-0.558	4.94	6	Nucleus

**Table 3:** Prediction of nuclear localization signals for identified C5-MTase and DMTase proteins in *A. cruentus*; cut off score = 2; protein with score >8 is predicted to be localized exclusively in nucleus, score >3 and <8 both the nucleus and the cytoplasm; score <2 localized in the cytoplasm.

Protein name	Monopartite NLSs	Starting position monopartite NLS	Score monopartite NLS	Bipartite NLSs	Starting position bipartite NLS	Score bipartite NLS
*AcMET1a*	–	–	–	3	10/224/224	2/2/2.3
*AcMET1b*	2	8/616	5/4	14	5/5/8/8/8/8/8/8/10/10/10/10/28/28	2.6/5.7/3.1/6.5/4.8/4.9/2.6/3.2/6.8/3.1/2.7/3.1/3.2/2.3
*AcCMT1*	1	16	2	12	12/14/14/14/14/14/16/16/16/18/18/18	2.5/7.2/3.6/7.1/2.3/4.2/6.4/2.6/4.4/6.2/3.7/3.4
*AcCMT2a*	3	85/626/628	2/6/3	1	19	2.5
*AcCMT2b*	2	187/937	3.5/2	2	77/934	3.2/3.3
*AcCMT3*	5	21/21/21/21/111	3/7/4.5/2/3.5	6	1/1/2/3/3/1280	6.1/4.9/4.3/5.1/3.7/2
*AcDRM2a*	2	243/246	12/6	6	233/243/246/246/246/495	4.5/4.5/6.5/2/2/2.8
*AcDRM2b*	1	170	5.5	2	171/421	7/3.6
*AcDML2a*	5	617/706/708/763/867	2.5/3/2/5/9	8	4/25/25/346/347/364/1894/1894	2/2/3.1/2/5.2/2/3.3/2.8
*AcDML2b*	1	12	2.6	–	–	–

**Figure 1:**
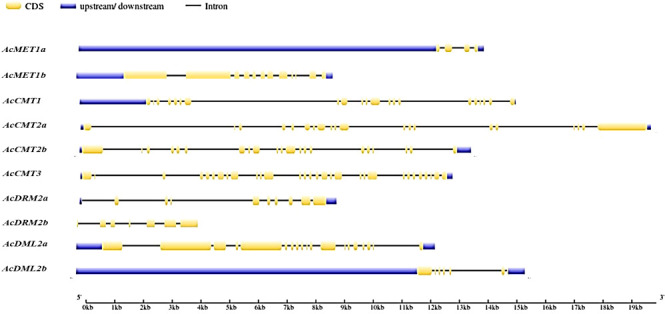
Gene structure of *A. cruentus C5-MTase* and *DMTase* genes; CDS regions are represented by yellow boxes; UTR regions by blues boxes; lines indicate intron regions.

The exon-intron architecture of the *A. cruentus MTase* and *DMTase* genes was characterized. The coding region of the *MTase* genes was interrupted by 3–29 introns, while that of the *DMTase* was interrupted by 6 and 18 introns, respectively. Among them, the *MTase* gene with the largest number of introns is AcCMT3, while AcMET1a contains the least introns (**Figure 1**).

### 3.2 Phylogenetic analysis

**Figure 2:**
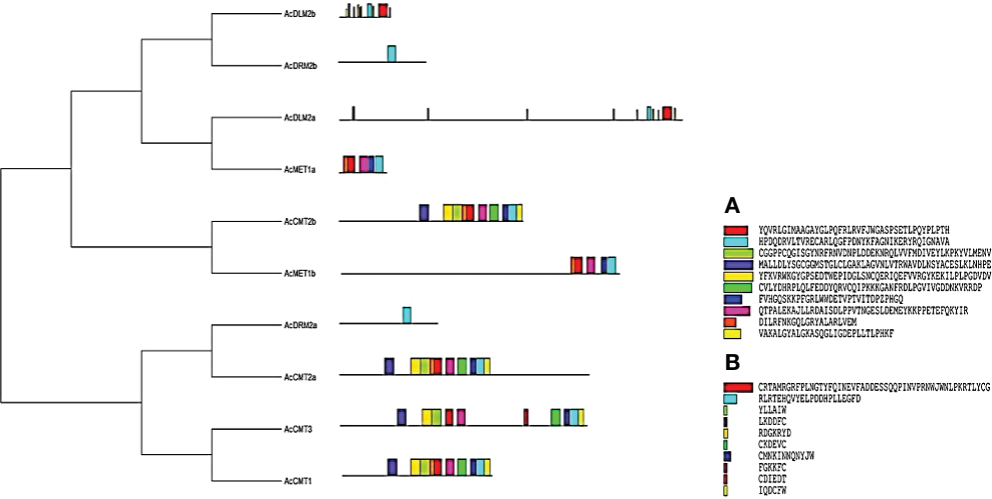
Evolutionary analysis and conserved motifs identified in *A. cruentus* C5-MTase and DMTase proteins; evolutionary analysis was performed using the Neighbor-Joining method; conserved motifs were identified using the MEME software, conserved motifs identified in **(A)** C5-MTases and **(B)** DMTases.

Evolutionary analysis was performed where *A. cruentus* C5-MTases and DMTase were divided into two main clusters. In total, 10 conserved motifs were identified for both C5-MTases and DMTase. The length of conserved motifs varied from 20 to 50 amino acids in analyzed C5-MTases and from 6 to 50 amino acids in DMTase (**Figure 2**).

### 3.3 Protein-protein interaction

The network of protein-protein interactions was computationally predicted using the STRING 11 database. The highest homology of identified C5-MTase and DMTase proteins was shown to *Beta vulgaris*. Therefore, the corresponding protein homologs of *A. cruentus* and *B. vulgaris* were aligned and an interaction map was constructed. Functionally, the analyzed proteins are involved in the following biological processes – DNA methylation on cytosine within a CG sequence, non-CG methylation, maintenance of DNA methylation, demethylation, base-excision repair and DNA metabolic process. Specifically, AcMET1a and AcMET1b showed 70 and 82% homology with *B. vulgaris* DNA (cytosine-5)-methyltransferase 1-b like isoform x1, respectively. A high homology was observed for all analyzed proteins, specifically AcCMT1 and putative DNA (cytosine-5)-methyltransferase CMT1 showed 71% identity. Furthermore, *A. cruentus* AcCMT2a, AcCMT2b and AcCMT3 share homology with *B. vulgaris* CMT2-like, CMT2 isoform x1 and CMT3 at levels 71, 72, and 77%, respectively. Both AcDRM2a and AcDRM2b were homologous to *B. vulgaris* DRM2-like at the level 70 and 82%. Amaranth demethylase AcDLM2a showed 64% homology with protein ROS1, and AcDLM2b with Demeter-like protein 3 isoform x1 has 49% homology.

**Figure 3:**
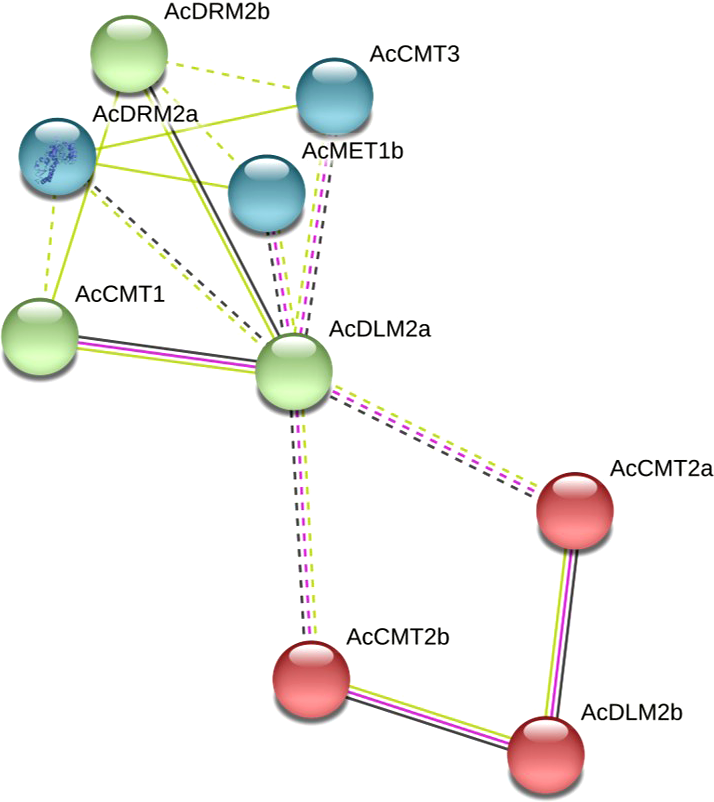
Computational prediction of protein-protein interaction network for AcC5-MTases and AcDMTases showing functional and physical associations among proteins. The dotted lines represent a relatively weak interaction while the solid lines indicate a relatively strong interaction. Colored lines between the proteins indicate the various types of interaction evidence: yellow line indicates textmining evidence, black line indicates coexpression evidence and purple line indicates experimental evidence.

The analyzed proteins were divided into three clusters at a confidence level of 0.40. The interaction among AcCMT2a, AcCMT2b and AcDLM2b was observed, then AcCMT1, AcDLM2a and AcDRM2b were clustered, and the third cluster was formed by AcDRM2a, AcMET1b and AcCMT3 (**Figure 3**). AcDLM2a and AcDLM2b interact relatively strongly with C5-MTases, mainly AcCMTs and AcDRM2b, suggesting that the level of cytosine methylation can be dynamically regulated by both C5-MTases and DMTases. 

### 3.4 *DNA MTase* and *DMTase* gene expression in *A. cruentus* in response to HM stress

**Figure 4:**
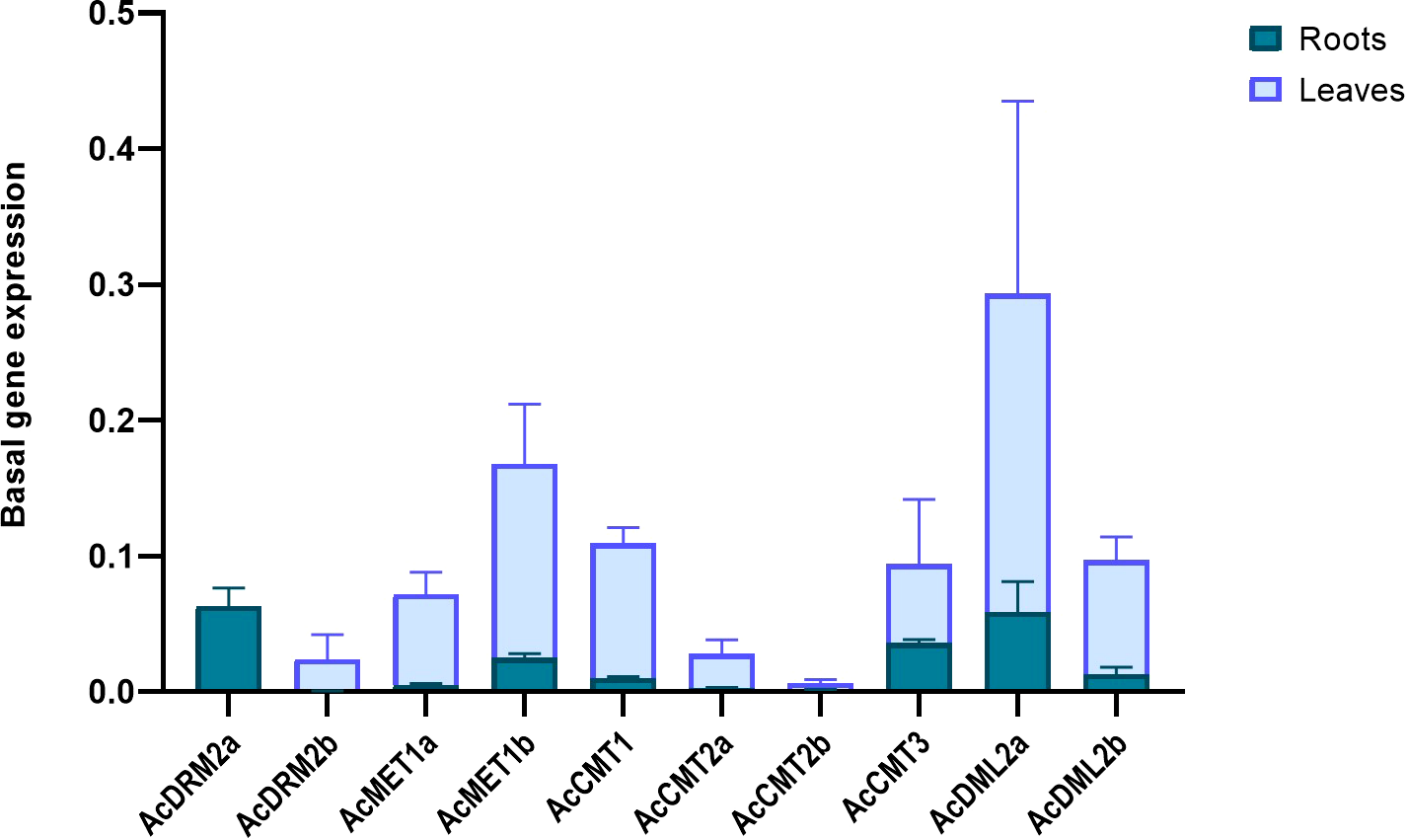
Basal expression of *A. cruentus MTase* and *DMTase* genes in root and leaf tissues in control conditions. Data were quantified using the 2−ΔΔCt method based on Ct values of *A. cruentus* MTase and DMTase genes and tubulin. Error bars indicate the mean ± SD (standard deviation) of three biological replicates.

**Figure 5:**
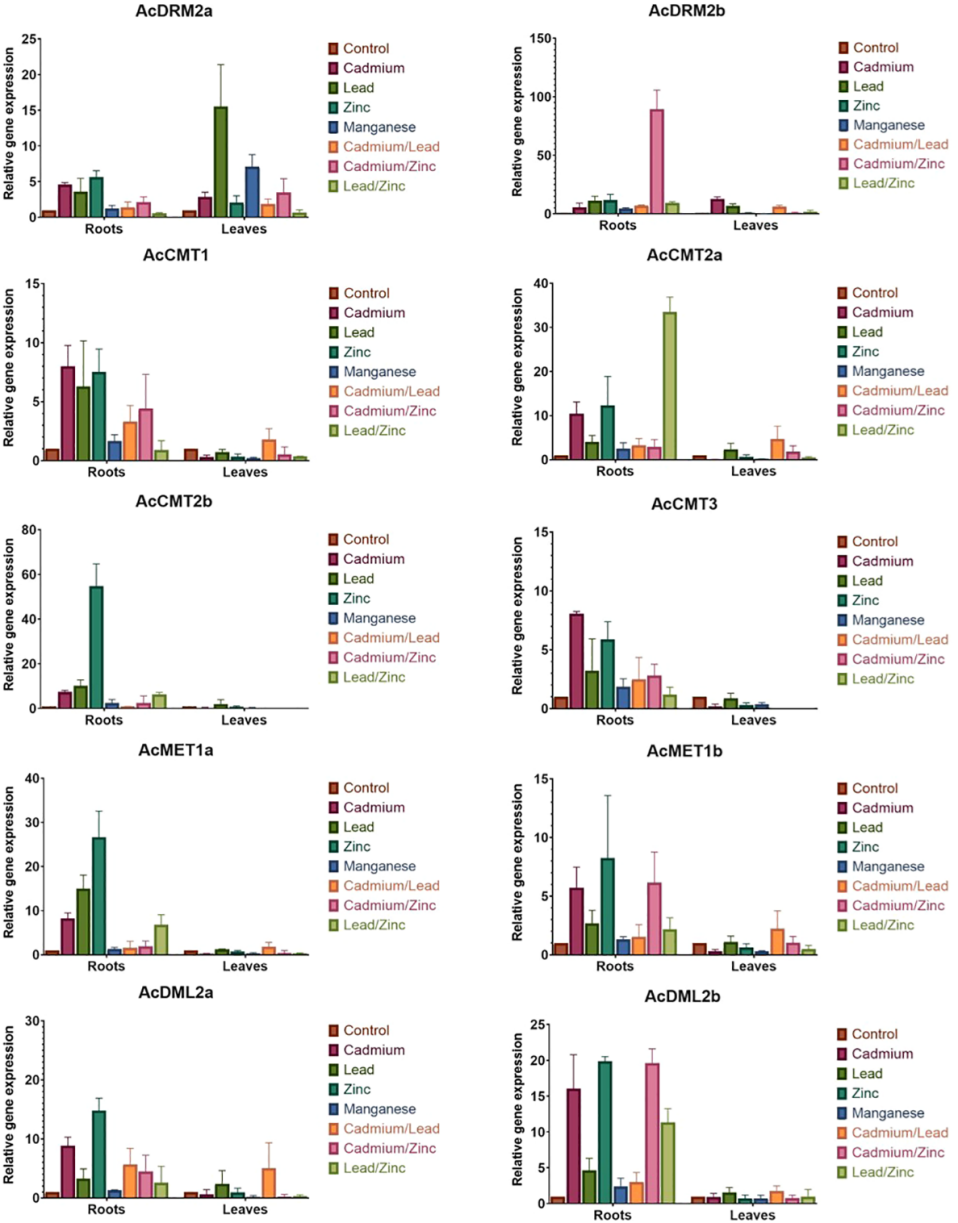
Relative expression of *A. cruentus MTase* and *DMTase* genes in root and leaf tissues under the individual and combined HM stress. Error bars indicate the mean ± SD (standard deviation) of three biological replicates. .

**Figure 6:**
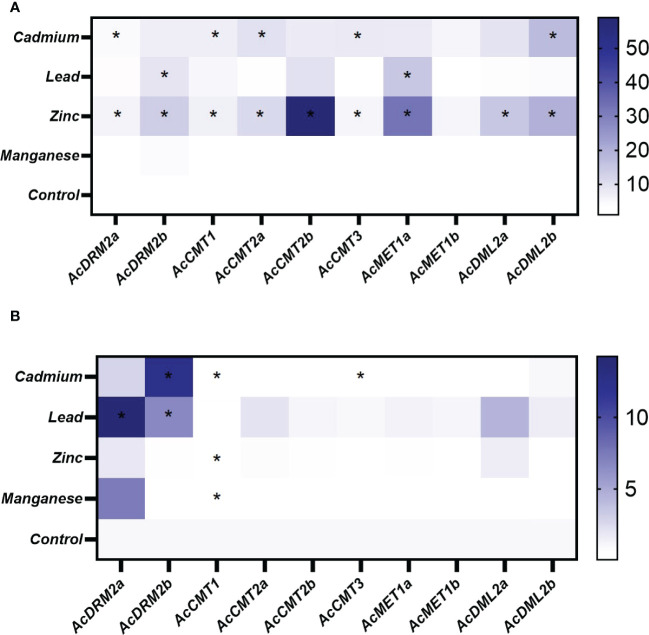
Heatmap representation of relative expression data for *A. cruentus MTase* and *DMTase* genes in the root **(A)** and leaf **(B)** tissues under the various HM stress; asterisks in heatmap show statistical significance at *p <*0.05 based on Tukey’s test when comparing HM-treated and control plants. The white and blue scale indicate relative expression to control samples where the expression level was set to 1.

Environmental pollution with HMs can significantly alter plant growth and development. Amaranth efficiently copes with HMs, even accumulates the large amount of metal ions into root tissues. However, the molecular mechanism of amaranth adaptive responses to HMs is still uncovered. To evaluate the responses of the *C5-MTase* and *DMTase* transcripts to metal stress, four metal ions - Cd, Pb, Zn, and Mn were applied in the excessive concentration. In general under the control conditions, *MTase* and *DMTase* genes showed higher expression in leaf tissues when compared to the roots (**Figure 4**). However, *de novo* MTase *AcDRM2a* gene expression was almost completely silenced in leaf tissues. Relative gene expression of *MTase* and *DMTase* genes in tested tissues under HMs exposure is shown in **Figure 5**. Significant changes in gene expression between control and HM-treated plants are schematically represented in a heatmap (**Figure 6**) based on the Tukey’s test.

When comparing the stress response in root and leaf amaranth tissues, notable gene response was primarily observed in roots (**Figure 5**). This is consistent with our hypothesis because roots are the first point of contact, and most HMs are stored in roots. When analyzing the effect of various HMs on the *MTase* and *DMTase* gene expression, Mn did not disturb the amaranth methylome. The expression of most genes was comparable to control plants in both tissues, with the exception of *AcDRM2a* and *AcCMT1*, which were differentially expressed in leaves. Interestingly, Zn triggered a much stronger stress response than toxic Cd. In response to Zn stress, almost all analyzed genes were significantly upregulated in roots (**Figure 6A**). Especially chromomethylase *AcCMT2b* showed 55-fold upregulation under this metal exposure. The effect of Cd exposure was manifested in root tissues by significantly higher activity of 3 chromomethylase genes (*AcCMT1*, *AcCMT2a*, and *AcCMT3*), *de novo MTase* gene *AcDRM2a* and *DMTase* gene *AcDML2b* (**Figure 6A**). The transcript level under Pb stress was significantly higher in *AcMET1a* and *AcDRM2b*, ensuring the maintenance of CG methylation and *de novo* DNA methylation.

As shown in **Figure 5**, *MTase* and *DMTase* gene expression in leaves was mostly steady or slightly upregulated in comparison to the controls. However, *de novo MTase AcDRM2a* and *AcDRM2b* were significantly upregulated in response to Cd and Pb. On the other side, *AcCMT1* was significantly downregulated in response to the Cd, Zn and Mn stress. Moreover, *AcCMT3* was significantly suppressed in response to the Cd stress (**Figure 6B**), which indicates the different regulation of DNA *MTases* depending on the metal stress.

### 3.5 Effect of combined HM stress on *MTase* and *DMTase* gene expression

**Figure 7:**
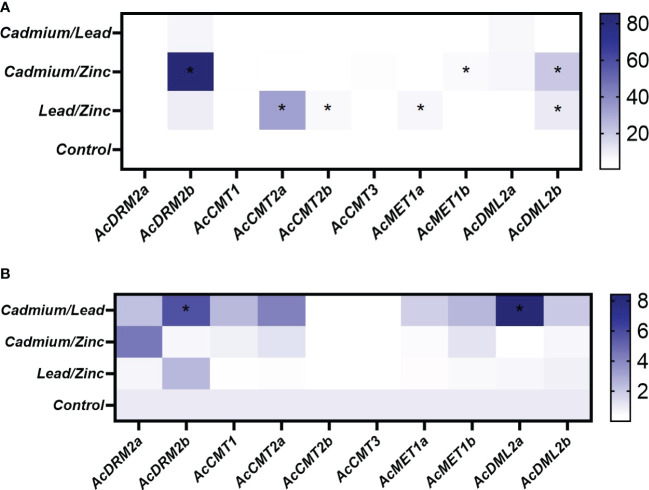
Heatmap representation of relative expression data for *A. cruentus MTase* and *DMTase* genes in the root **(A)** and leaf **(B)** tissues under the combined HM stress; asterisks in heatmap show statistical significance at *p <*0.05 based on Tukey’s test when comparing HM-treated and control plants. The white and blue scale indicate relative expression to control samples where the expression level was set to 1.

The expression pattern of *MTase* and *DMTase* genes was also investigated under combined HM stress (**Figures 5**, **7**). As well studied toxic metals, Cd and Pb ions and their combination were applied. Additionally, Zn was used as an essential metal that showed the most notable effect on expression patterns of investigated *MTase* and *DMTase* genes, mainly in roots (**Figure 6A**).

Interestingly, the combination of two toxic metals Cd/Pb did not significantly upregulate gene expression in below-ground tissues (**Figure 7A**). Unlike roots, combined Cd/Pb stress triggered the most significant response in leaf tissues. Specifically, *de novo MTase* gene *AcDRM2b* and DMTase *AcDML2a*, were significantly upregulated 6 and 5-fold, respectively (**Figure 7B**). Unexpectedly, the expression of *AcCMT2b* and *AcCMT3* was completely silenced in leaves during this combined HM stress.

However, there is clear evidence that combined stress involving Zn led to the significant response of specific *MTase* and *DMTase* genes. In roots, genes *AcMET1b* and *AcDRM2b*, responsible for the maintenance of CG methylation, were significantly (6 and 90-fold, respectively) upregulated in response to the simultaneous Cd/Zn stress (**Figure 7A**). Furthermore, DMTase gene *AcDML2b* showed 20-fold upregulation. As the results show, the *AcDRM2b* appears to play a crucial role in response to the Cd/Zn stress, perhaps protecting the amaranth genome from excess stress by hypermethylation. At the same time, DMTase *AcDML2b* counteracts uncontrolled DNA methylation.

DMTase gene *AcDML2b* was also significantly upregulated in response to the combined Pb/Zn stress, which suggests its involvement in genome protection against hypermethylation. Simultaneous Pb/Zn exposure induced significant upregulation of *AcMET1a* and chromomethylases *AcCMT2a* and *AcCMT2b* (**Figure 7A**).

## 4 Discussion

In the last few years, relatively forgotten pseudocereal amaranth (*Amaranthus* spp.) gained the interest of researchers worldwide. When climate change and environmental pollution are in focus, amaranth’s adaptability and tolerance to harsh environmental conditions become interesting. These attributes might also be important for agricultural purposes and soil remediation strategies (Li et al., 2016; Guo et al., 2019; Wang et al., 2019; Lancíková et al., 2020; Njoku and Nwani, 2022). However, there is little known about amaranth stress response at the molecular level. Based on our knowledge, no study dealing with epigenetic regulation of stress response has been performed up to this date.

Herein, eight *A. cruentus C5-MTase* and two *DMTase* genes were identified. Three groups of *C5-MTase* genes, MET, CMT and DRM, were recognized. As regards to *MET* genes, analysis resulted in identification of two genes, similar to soybean or rice (Sharma et al., 2009; Garg et al., 2014). In comparison, there are six members of MET group in rapeseed and only one in tomato or pea (Pavlopoulou and Kossida, 2007; Cao et al., 2014; Fan et al., 2020a). Moreover, four members of the CMT group were discovered in amaranth and also in soybean, while there are three members in rice and six members in rapeseed (Sharma et al., 2009; Garg et al., 2014; Fan et al., 2020a). Amaranth harbors *CMT1* gene also identified in *Arabidopsis*, however, concluded as defective and/or silent. Interestingly, globe artichoke lacks CMT1 homolog (Ashapkin et al., 2016). Likewise in this study, all rapeseed CMT proteins were also predicted to be localized in the cell nucleus (Fan et al., 2020a), while the predicted localization of the other MTases varied. Only two *DRM* genes were recognized in *A. cruentus*, while rice, soybean or rapeseed contain four, five, and eight *DRM* genes, respectively (Sharma et al., 2009; Garg et al., 2014; Fan et al., 2020a).

From the two DMTase groups, only members of the DML2 group have been found in *A. cruentus*. DMTases DML2 belong to the 5-methylcytosine DNA glycosylases, which are expressed in many plant organs and are required to remove DNA methylation marks from improperly-methylated cytosines, but also to maintain high levels of methylation in properly targeted sites (Ortega-Galisteo et al., 2008). In the *Arabidopsis* genome, all groups of *DMTase* genes, including DML2, are encoded (Schumann et al., 2017). Also, higher number of the DME, DML3 and ROS1 groups have been discovered and identified in rapeseed than that of *A. cruentus* (Fan et al., 2020a).

Structural analysis showed that MET1 in *A. cruentus* is characterized by presence of two RFD and two BAH domains likewise observed in *Solanum melongena* (Kumar et al., 2016). It is assumed that one BAH domain mediates MET1 interaction with histone tails and second BAH domain ensures interaction with other proteins (Garg et al., 2014). The amaranth DMTases possess Perm-CXXC, which is the main attribute of Demeter-like proteins in plants (Gianoglio et al., 2017). Moreover, RRM-DME, ENDO3c belonging to the HhH-GPD domain and iron-sulphur binding FES domain which belongs to the ENDO3 superfamily. Specifically, RRM-DME sequence consists of 90 residues identified in RNA and ssDNA-binding proteins. The domain HhH-GPD is linked to base excision repair DNA glycosylase (Gianoglio et al., 2017). Protein-protein interaction analysis revealed that DMTases interact relatively strongly with C5-MTases, especially AcCMTs and AcDRM2b. Similar results were obtained in the study by Yu et al. (2021), in which it was supposed that C5-MTases and DMTases may form a reciprocal negative feedback loop that dynamically affects the overall level of cytosine methylation.

A close relationship exists between physiological responses, gene expression levels, and DNA methylation patterns under HM stress. Hypermethylation is considered one of the defense strategies of plants to protect against possible damage by HM products (Sun et al., 2022). Modulation of the metal stress response by DNA methylation has been reported in many different species, including important cereals such as wheat and barley (Kong et al., 2020). It is assumed that different plant protection mechanisms may exist depending on whether the heavy metal element is essential for plant growth or not (Sun et al., 2022). In the present study, DNA methylation also varied within the combination of metals as well as with individual metal application.

We analyzed the transcript abundance of identified *MTase* and *DMTase* genes under exposure to four metals (Cd, Pb, Zn, Mn) and three of their combinations (Cd/Pb, Cd/Zn, Pb/Zn). Our results indicate that one MTase/DMTase gene could be regulated by more than one metal. Similarly, Shafiq et al. (2020) concluded their expression study of MTases in maize under the same metal treatments.

Our previous results demonstrated uptake and accumulation of significant portion of Cd mainly into roots, with low translocation into aerial parts (Lancíková et al., 2020). Consistent with previous findings, Cd induced significant upregulation of five genes in roots, including one DMTase, while only one gene was upregulated in shoots (*AcDRM2b*). However, the activity of two upregulated genes (*AcCMT1* and *AcCMT3*) in roots was significantly suppressed in shoots. This indicates that level of Cd-induced stress decreased in aerial plant parts. These chromomethylase genes are strongly associated with non-CG methylation (Stroud et al., 2014; Ashapkin et al., 2020). In leaves, only Ac*DRM2b* gene was significantly responsive to Cd and Pb stress either when applied individually or in Cd/Pb combination. However, when these metals were applied with Zn, the Ac*DRM2b* transcript was identified in leaf tissues as low. As for the amaranth roots, *DRM* genes were upregulated in response to the Cd, Pb or Zn stress and in Cd/Zn combination. Type of DRM proteins is present solely in plants. In chickpea roots, *DRM* genes were upregulated in response to drought, cold and salt stress (Garg et al., 2014). Differential expression of DNA MTases in response to Pb, Cd and Zn metal treatment was also observed in wheat and maize (Shafiq et al., 2019; Shafiq et al., 2020).

Sun et al. (2021) indicated that soybean resists Cd stress *via* an increased level of genomic DNA methylation, with the methylation level increasing with increased Cd concentration. Similarly, increased Cd concentrations combined with Mn boosted the number of differentially methylated loci in pokeweed (Jing et al., 2022). On the other hand, Cd-exposed and Cd-free rice plants had similar genomic cytosine methylation levels and no difference in DNA methylation marks was observed between the roots and shoots of rice seedlings exposed to Cd (Feng et al., 2016).

In response to individual Pb, amaranth roots showed significant upregulation of *AcMET1a* gene, responsible for maintenance of CG methylation, and *AcDRM2b* gene, responsible for *de novo* non-CG methylation (Brocklehurst et al., 2018). *AcMET1* was upregulated in the case of combined Cd/Zn and Pb/Zn stress, while *AcCMT* was upregulated only in the case of Pb/Zn. Apart from *MET1* and *CMT* genes also *DML2b* gene showed increased expression in root tissue under this metal combination. Specific changes in DNA methylation were also observed in response to the combination of metals (Pb, Cd and Zn) in maize. This suggests that the combination of metals could produce different levels of DNA methylation compared to individual metals (Shafiq et al., 2020).

Zn-induced stress significantly increased transcript abundance in amaranth root tissues in almost all analyzed *MTase* and *DMTase* genes. In contrast, the expression of studied genes was mainly comparable to the controls under Mn stress. Moreover, *AcCMT1* was significantly downregulated in aerial parts in response to Zn and Mn stress. The amaranth genome encodes three types of *CMT* genes, *CMT1*, *CMT2* and *CMT3*, which is comparable to the *Arabidopsis* genome. *CMT1* is the least studied gene because it has been suggested that *CMT1* is non-essential (Bewick et al., 2017). Stroud et al. (2014) tested CMT2 and CMT3 activity *in vitro* and confirmed their essential involvement in CHH and CHG methylation. Interestingly, CMT2 primarily methylated unmethylated sequences in both CHH and CHG sites while CMT3 preferentially targeted CHG sites at the hemimethylated sequences. Nevertheless, CMT3 is widely associated with non-CG methylation, there is a link between CMT3 and CG context gene-body methylation observed in Brassicaceae species (Bewick et al., 2017).

In control conditions, *MTase* genes showed higher expression in leaf tissues compared to roots. On the other hand, under HM stress, the expression of *MTase* genes was higher in root tissues. The exception was *AcDRM2a* gene, which had a higher expression in the roots under control conditions and in leaves under HM stress, especially under Pb stress. In general, none of the *AcCMTs* and *AcMETs* genes were significantly upregulated in leaves under HM stress. Some of them were significantly downregulated and AcCMT2b and AcCMT3 were completely silenced under combined HM stress in leaves. Given that the roots are in contact with heavy metals, we assume that DNA methylation occurs mainly in this tissue. The pattern of DNA methylation is organized by the interplay of DNA methylation and demethylation. In case of acute stress, hypermethylation can be balanced by demethylation process which prevents transposable elements from accumulation of DNA methylation. As a result, DNA methylation is not spreading into adjacent genes (Penterman et al., 2007; Parrilla-Doblas et al., 2019). Demethylation has also been found in response to HMs (Sun et al., 2022).

Upregulation of *DML2* DMTases genes was observed in amaranth root tissues in response to Cd and Zn stress either when applied individually or in combination. DMTase gene *AcDML2b* was also significantly upregulated in response to the combined Pb/Zn stress in roots and *AcDML2a* was significantly upregulated in response to the combined Cd/Pb stress in aerial tissue. Similar observation, thus upregulation of DMTases, was described in eggplant in response to salt and drought stress (Moglia et al., 2019). In rapeseed, *DMTase* genes were up- or down-regulated in response to hot and salt stress (Fan et al., 2020a). In the study by Gu et al. (2016), expression of some *DMTase* genes increases in response to various abiotic stresses including heat, cold, drought and salinity in *Fragaria vesca*. On the other hand, *DMTase* genes were significantly inhibited and DNA methylation was increased at the genome-wide level in *Arabidopsis* plants under Cd stress (Fan et al., 2020b).

## 5 Conclusion

Eight *C5-MTase* (*MET*, *CMT* and *DRM*) and two *DMTase* (*DML2*) genes were discovered and identified in *A. cruentus* genome. Phylogenetic analysis separated identified genes into two main clusters. The expression pattern of identified genes varies in response to individual and combined metal treatments. Not surprisingly, the response to HM stress was observed mostly in roots compared to leaves, since the roots are the first point of contact and studied HMs are primarily stored in the roots. Further, transcripts of identified genes were highly abundant under Zn treatment, suggesting they are implicated in the mechanisms to protect the amaranth plant from Zn-induced stress. The results, however, document the involvement of *C5-MTase* and *DMTase* in general response to heavy metal-induced stress. Several other studies have also shown that patterns in methylation and demethylation levels differ by type of HM, but the results of some studies are contradictory. This suggests that HM-induced DNA methylation and demethylation in plants is a rather complicated process that requires further investigation.

## Data availability statement

The original contributions presented in the study are included in the article/supplementary material. Further inquiries can be directed to the corresponding author.

## Author contributions

VL and AH conceived the experiment. VL performed the experiment, analyzed the data and wrote the manuscript. JK analyzed the data and wrote the manuscript. AH reviewed and edited the manuscript. All authors contributed to the article and approved the submitted version.
